# Recent Advances in the Treatment of Malaria

**DOI:** 10.3390/pharmaceutics16111416

**Published:** 2024-11-04

**Authors:** Jawaher M. Alghamdi, Arwa A. Al-Qahtani, Fatimah S. Alhamlan, Ahmed A. Al-Qahtani

**Affiliations:** 1 Department of Zoology, College of Science, King Saud University, Riyadh 13242, Saudi Arabia; jaalghamdi@ksu.edu.sa; 2 Department of Family Medicine, College of Medicine, Al-Imam Mohammad Ibn Saud Islamic University, Riyadh 11432, Saudi Arabia; arahalqahtani@imamu.edu.sa; 3 Department of Infection and Immunity, King Faisal Specialist Hospital & Research Center, Riyadh 11211, Saudi Arabia; falhamlan@kfshrc.edu.sa; 4 Department of Microbiology and Immunology, College of Medicine, Alfaisal University, Riyadh 11211, Saudi Arabia

**Keywords:** resistance, malaria, malaria treatment, artemisinin-based combination therapies (ACTs), vaccines, triple artemisinin combination therapies (TACTs)

## Abstract

Malaria is still one of the major global health challenges affecting millions annually, particularly in non-Mediterranean Africa and Southeast Asia. Over the past two decades, substantial progress has been made in reducing malaria-related morbidity and mortality, primarily due to advancements in antimalarial therapeutics. This review provides a comprehensive overview of recent developments in malaria treatment, focusing on the evolution of drug therapies, mechanisms of action, and emerging resistance patterns. The cornerstone of current treatment strategies is artemisinin-based combination therapies (ACTs), which have proven highly effective against *P. falciparum* and *P. vivax*, the most prevalent malaria-causing parasites. However, the onset of artemisinin resistance, particularly in Southeast Asian countries, poses a significant threat to these gains. Additionally, other antimalarial classes, including quinine derivatives, 8-aminoquinolines, and antifolate drugs, are examined for their efficacy, resistance mechanisms, and future potential. This review also discusses the challenges associated with drug resistance, the genetic underpinnings of resistance in malaria parasites, and the implications for future treatment protocols. Furthermore, the review examines combinational therapies, such as triple artemisinin combination therapies (TACTs), and vaccines that are approved or in development to circumvent resistance issues. The need for continuous surveillance, innovative therapeutic strategies, and advances in novel antimalarial therapeutic agents is emphasized to sustain and further progress in the control of malaria and its eventual eradication.

## 1. Introduction

Malaria is still one of the major global health challenges affecting millions of people annually predominantly in non-Mediterranean Africa, South Asia, and parts of Latin America [1]. According to the World Health Organization (WHO), approximately 241 million malaria-related cases have been reported, out of which 627,000 succumbed to death in 2020 alone, with children under five being particularly vulnerable [2]. This parasitic infection is caused by *Plasmodium* species, especially *Plasmodium falciparum* and *Plasmodium vivax*, which are transmitted through the bites of infected Anopheles mosquitoes.

There are more than 120 known *Plasmodium* species, of which only five are responsible for causing malaria in humans: *P. falciparum*, *P. vivax*, *P. ovale*, *P. malariae*, and *P. knowlesi*. *P. falciparum*, the deadliest of the lot, is responsible for over 90% of all malaria-related deaths worldwide [3,4]. Geographically, *P. falciparum* is a major etiological factor for the vast majority of malaria cases in African regions, being responsible for over 99% of infections. It also poses a significant problem in other regions, including the Western Pacific (71.9%), the Eastern Mediterranean (69%), and Southeast Asia (62.8%) [5]. Although *P. vivax* is typically associated with uncomplicated malaria, there is evidence that it can also lead to severe illness [6]. *P. knowlesi*, a zoonotic parasite transmitted from primates to humans, can also cause severe malaria [7]. *P. malariae* and *P. ovale* generally result in uncomplicated malaria but can occasionally be linked to additional complications [8]. The lifecycle of the Plasmodium parasite is complex, involving both human and mosquito hosts. In humans, the parasite undergoes a liver stage followed by a blood stage where it infects red blood cells, leading to clinical manifestations of malaria, such as fever, chills, and anemia [4]. Understanding this lifecycle has been crucial in developing targeted interventions and treatments.

Historically, malaria treatment has evolved from the use of natural remedies, such as quinine derived from the bark of the cinchona tree, to synthetic drugs like chloroquine and sulfadoxine–pyrimethamine [9]. However, the development of drug resistance has necessitated continuous innovation in antimalarial therapies. The introduction of artemisinin-based combination therapies (ACTs) in the early 2000s marked a significant breakthrough, offering a highly effective treatment for uncomplicated malaria and helping to reduce mortality rates [9]. Despite significant progress, the control and eventual eradication of malaria face numerous challenges. The major pressing issue is the frequent reduction in the efficacy of drugs due to resistance caused by *Plasmodium* strains. Resistance to first-line treatments, including ACTs, has been documented, mainly in the subregion of Greater Mekong (GM) [10]. This resistance threatens to undermine the gains made in malaria control and highlights the urgent need for new and effective antimalarial drugs. In addition to drug resistance, other challenges include the high cost of drug development, regulatory hurdles, and the need for efficacious drugs for exposed populations, including pregnant women and young children [11,12]. The geographical variation in *Plasmodium* species and their resistance patterns also complicates treatment strategies.

The importance of ongoing drug discovery and development cannot be overstated. New therapies must not only address the issue of resistance but also offer improved efficacy, safety, and accessibility. Advances in our understanding of the parasite’s biology and host interactions, coupled with innovative technologies in drug discovery, hold promise for the development of next-generation antimalarial agents. This review offers a detailed overview of recent advances in the therapies of malaria, accompanied with a particular focus on drug discovery and development. It will examine novel drug targets, innovative screening methods, and the advancement of novel treatment modalities for malaria. Additionally, the review will explore the challenges faced in the field and discuss future directions for research and development. By highlighting these recent efforts, the review seeks to contribute to the ongoing quest for effective and sustainable malaria treatments, ultimately supporting global malaria eradication efforts.

## 2. Current State of the Art in Malaria Treatment Modalities

### 2.1. Quinine Derivatives

Quinine derivatives, including chloroquine, amodiaquine, lumefantrine, halofantrine, and mefloquine, are critical components in the treatment of malaria. These compounds have been developed and utilized primarily due to their efficacy in combating the *P. falciparum* parasite [13]. They are often used in combination therapies to enhance treatment effectiveness and reduce the risk of drug resistance. These compounds are characterized by their chemical structure, which typically includes an aromatic ring connected to an amino alcohol moiety, contributing to their antimalarial activity.

#### 2.1.1. Mechanisms of Action

The primary mechanism of action of quinine derivatives involves interference with the parasite’s ability to detoxify heme, a byproduct of hemoglobin digestion [14]. During the intraerythrocytic phase of its lifecycle, *Plasmodium* species degrade hemoglobin to acquire amino acids, thus releasing free hematin, which is noxious to the parasite. To mitigate this toxicity, the parasite converts free heme into a non-toxic form called hemozoin. The quinine derivatives are believed to bind to free heme or to the heme polymerization process, preventing its detoxification and leading to the accumulation of toxic heme inside the parasite. This disruption in heme detoxification ultimately results in parasite death. Additionally, these compounds may also impair the function of the parasite’s membranes and inhibit nucleic acid synthesis [15].

#### 2.1.2. Treatment Modalities

A quinine derivative compound is often utilized in combination with other antimalarial drugs to form combination therapies, which are the cornerstone of modern malaria treatment strategies. For instance, lumefantrine is commonly paired with artemether in the fixed-dose combination known as Coartem [16]. This combination leverages the rapid action of artemether with the longer half-life of lumefantrine to provide sustained antimalarial action and decrease the recrudescence risk. Similarly, mefloquine has been utilized both as a monotherapy and in combination with other medications, including artesunate, to treat multidrug-resistant malaria [10]. These combination therapies are designed to enhance efficacy, reduce the duration of treatment, and minimize the development of resistance.

### 2.2. 8-Aminoquinoline Compounds

8-Aminoquinoline compounds, such as primaquine and tafenoquine, are classified as antimalarial medicines primarily used for their unique ability to target plasmodium exoerythrocytic stages (liver stage), including the dormant hypnozoites of *P. vivax* and *P. ovale* [17]. These compounds are essential in the eradication therapy of recurring malaria, a form of the disease where the parasites can lie dormant in the liver and reactivate weeks or months after the initial infection. Their discovery and development have significantly improved the treatment and management of these persistent forms of malaria.

#### 2.2.1. Mechanisms of Action

The exact mechanisms of action of 8-aminoquinolines are not fully understood, but several hypotheses exist. These compounds are thought to interfere with the parasite’s mitochondrial function and disrupt its electron transport chain, leading to reactive oxygen species (ROS) generation [18]. The oxidative stress induced by ROS damages cellular components, leading to parasite death. Additionally, 8-aminoquinolines may also interfere with the parasite’s DNA and protein synthesis [19]. Their ability to target both parasitic stages (liver and blood) renders them invaluable property to treat malaria.

#### 2.2.2. Treatment Modalities

8-Aminoquinolines are primarily used for the radical cure and prevention of relapses in *P. vivax* and *P. ovale* malaria. Primaquine has been used as a standard of cure for many years, being typically co-administered with other antimalarial medications to clear the blood-stage parasites [20,21]. The usual course of primaquine involves a daily dose for 14 days. Tafenoquine, a newer 8-aminoquinoline, has the advantage of a longer half-life, allowing for a single-dose treatment regimen, which improves patient compliance and reduces the likelihood of incomplete treatment [17,22]. In addition to their role in treating relapsing malaria, 8-aminoquinolines are also used in malaria prophylaxis. Primaquine is used to prevent malaria in travelers to endemic areas, particularly where *P. vivax* and *P. ovale* are prevalent [23]. Tafenoquine has also been approved for prophylactic use, providing a convenient option for travelers due to its once-weekly dosing schedule [24].

### 2.3. Antifolate Compounds in Malaria Treatment

Antifolate compounds are fundamental to antimalarial action and have significantly contributed to the fight against malaria. These drugs inhibit the folate pathway, which is essential for DNA synthesis and cell division in Plasmodium parasites [25]. Antifolate drugs have been particularly useful in areas with chloroquine-resistant malaria and are often used in combination therapies to enhance efficacy and reduce resistance [26]. Despite their effectiveness, the rise of drug-resistant strains presents ongoing challenges.

#### Mechanisms of Action

Antifolate compounds function by targeting the folate biosynthesis pathway, which is essential for the production of the nucleotides required for DNA and RNA synthesis in Plasmodium parasites [27]. These drugs primarily inhibit two key enzymes within this pathway. The first target, dihydrofolate reductase (DHFR), is responsible for reducing dihydrofolate to tetrahydrofolate, a critical reaction in folate metabolism. The inhibition of DHFR by antifolate drugs such as pyrimethamine and proguanil prevents the formation of tetrahydrofolate, thereby disrupting DNA synthesis and cell division [28]. The second target is dihydropteroate synthase (DHPS), an enzyme involved in the early stages of folate synthesis. DHPS catalyzes the formation of dihydropteroate from para-aminobenzoic acid (PABA). Sulfadoxine inhibits DHPS, reducing the availability of folate precursors and further impairing DNA synthesis [28]. By blocking these enzymes, antifolate compounds effectively disrupt the folate pathway, leading to the death of the parasite.

Antifolate drugs are integral components of various antimalarial treatment regimens and are often combined with other antimalarials to boost efficacy and mitigate resistance [29]. One prominent combination is sulfadoxine–pyrimethamine (SP), which is widely used to treat uncomplicated malaria caused by *P. falciparum* [30]. SP is also employed as an alternative therapy for prevention in pregnant women and infants, providing crucial resistance towards malaria during these vulnerable stages. Another significant antifolate is proguanil, which is frequently combined with atovaquone, a hydroxy-1,4-naphthoquinone, in the formulation known as Malarone [31]. In this combination, proguanil enhances the efficacy of atovaquone by inhibiting DHFR, making it effective for both treatment and prophylaxis of malaria, especially in regions with multidrug-resistant strains.

Chlorproguanil, historically used in combination with dapsone under the name Lapdap, showcased the utility of combining antifolate drugs with other antimalarials to enhance their effectiveness [32]. However, due to safety concerns, chlorproguanil has been withdrawn from many markets [33]. Nonetheless, its use underscored the potential of such combinations in malaria treatment strategies. Additionally, trimethoprim, an antibacterial, is sometimes repurposed in combination with sulfamethoxazole as co-trimoxazole. This combination is used for malaria prophylaxis in specific populations, such as individuals living with HIV/AIDS, demonstrating the versatility and broad applicability of antifolate drugs in combating malaria [34].

### 2.4. Artemisinin Compounds

Artemisinin compounds, derived from the sweet wormwood plant (*Artemisia annua*), represent one of the most significant breakthroughs in the treatment of malaria. These compounds, including artemisinin itself and its derivatives such as artesunate, artemether, and dihydroartemisinin, are known for their rapid action and high efficacy against *Plasmodium* species, particularly *P. falciparum*. ACTs have become the cornerstone of malaria treatment worldwide, advised by the WHO as an ideal therapeutic option for uncomplicated cases of malaria [21].

#### 2.4.1. Mechanisms of Action

The primary functional mechanism of artemisinin compounds involves ROS generation inside the parasite [35]. When artemisinin interacts with heme or intracellular iron, it undergoes the breakdown of its endoperoxide bridge, leading to the formation of free radicals. These radicals cause oxidative damage to parasite proteins, lipids, and membranes, resulting in parasitic demise [35,36]. Additionally, artemisinin compounds inhibit essential parasite enzymes and disrupt various cellular processes, including mitochondrial function and calcium homeostasis [37]. The multifaceted attack on the parasite makes it difficult for resistance to develop, although resistance has been emerging in some regions.

#### 2.4.2. Current ACT Treatment Modalities

ACTs have revolutionized the treatment of malaria, particularly *P. falciparum* infections. Among these, artemether–lumefantrine (Coartem), artesunate–amodiaquine, dihydroartemisinin–piperaquine (DHA-PPQ), and artesunate–mefloquine are prominent combinations, each with unique pharmacological properties and clinical applications [38].

##### Artemether–Lumefantrine (Coartem)

Artemether–lumefantrine (*Coartem*) is among the predominantly used ACTs globally, particularly for uncomplicated *P. falciparum* malaria. The combination leverages the rapid action of artemether and the longer half-life of lumefantrine to ensure both immediate and sustained antimalarial effects [39]. A systematic review study involving 8320 patients indicated significant effectiveness of artemether–lumefantrine, with a pooled PCR (polymerase chain reaction)-corrected adequate clinical and parasitological response (ACPR) rate of 97% at day 28. Early treatment failures were nearly absent, and late treatment failures were reported at less than 8% [40]. Rapid parasite clearance was observed, with over 93% of patients being parasite-free by day three of treatment. Artemether–lumefantrine is administered in a fixed-dose combination, typically six doses over three days, with food enhancing its absorption. The standard regimen for adults is 24 tablets over three days, with specific dosages adjusted for pediatric patients based on weight [41].

While generally well tolerated, artemether–lumefantrine can cause prolongation of QT, a measure of the time it takes for the heart’s electrical system to reset after each heartbeat, necessitating caution in patients with pre-existing heart conditions [42]. Additionally, it may reduce the effectiveness of hormonal contraceptives, prompting recommendations for alternative contraceptive methods during treatment.

##### Artesunate–Amodiaquine

Artesunate–amodiaquine is another effective ACT, being particularly prevalent in Africa [43]. Artesunate provides rapid parasite clearance, while amodiaquine contributes to the sustained suppression of the parasite [44]. Clinical studies have demonstrated that artesunate–amodiaquine is effective in treating uncomplicated malaria, with a favorable safety profile. The combination has been associated with low rates of treatment failure and is particularly beneficial in regions where resistance to other antimalarials is prevalent [40]. The combination is typically administered over three days, similar to other ACTs, with specific dosing guidelines based on patient age and weight. This regimen is effective in ensuring rapid and sustained antimalarial action.

##### Dihydroartemisinin–Piperaquine (DHA-PPQ)

Dihydroartemisinin–piperaquine is notable for its dual role in both the treatment and prophylaxis of malaria. Dihydroartemisinin acts rapidly, while piperaquine extends the duration of action due to its long half-life. DHA-PPQ has shown high efficacy in various studies, with significantly high cure rates in uncomplicated malaria cases [45]. Its prolonged action makes it suitable for both treatment and potential prophylactic use in high-risk populations [46]. The safety profile of DHA-PPQ is generally favorable, with few adverse effects reported [45,46]. It is particularly advantageous in areas with high malaria transmission rates, where sustained protection is essential.

##### Artesunate–Mefloquine

Studies have demonstrated the effectiveness of artesunate–mefloquine in treating multidrug-resistant falciparum malaria [47,48,49]. An earlier randomized trial conducted on the Thailand–Myanmar border compared artesunate–mefloquine, artemether–mefloquine, and mefloquine alone [50]. The artesunate and artemether combinations showed very similar clinical and parasitological responses, with significantly shorter fever and parasite clearance times compared to mefloquine alone. After adjusting for reinfections, the failure rates were 13.9% for the artesunate combination and 12.3% for the artemether combination compared to 49.2% for mefloquine alone (*p* < 0.0001). Another study in Africa assessed the non-inferiority of artesunate–mefloquine to artemether–lumefantrine in uncomplicated cases of *P. falciparum* malaria in young children. The PCR-driven ACPR rate at 63 days was 90.9% for artesunate–mefloquine and 89.7% for artemether–lumefantrine, demonstrating non-inferiority. Artesunate–mefloquine also delayed the time to reinfection compared to artemether–lumefantrine [51]. The artesunate–mefloquine combination is administered over three days, with careful monitoring for potential side effects, including neuropsychiatric effects associated with mefloquine [52]. Patients are advised to be aware of these risks, particularly if they have a history of psychiatric disorders.

## 3. Genetic Basis for Resistance to Antimalarials

Antimalarial agents primarily target the blood-associated, non-sexual stages of parasitic malaria, which cause the disease’s symptoms (Table 1) [53]. The two main parasites, *P. falciparum*, and *P. vivax*, have developed resistance to almost all antimalarial drugs, exemplifying the one most significant setback to the use of antimalarials. In *P. falciparum*, chloroquine resistance arises from point mutations in genes encoding chloroquine resistance transporter (*pfcrt*) and P-glycoprotein transporter proteins (*pfmdr*), reducing drug accumulation in the parasite’s digestive vacuole [54]. Detecting resistance to chloroquine in *P. vivax* is more difficult due to lower parasitemia and the challenge of distinguishing between residual negative effects and disease relapse [55]. The lack of a reliable molecular marker for chloroquine resistance in *P. vivax* poses significant challenges for monitoring this growing concern. The *pfcrt* analog, *P. vivax* chloroquine resistance transporter-o (*pvcrt*-o), exhibits mutational and chloroquine resistance [56]. Although a previous study in the Brazilian Amazon found that increased copies of the gene encoding *pvcrt*-o are associated with chloroquine resistance in recurrent vivax infections, a more recent study in a Malaysian cohort exhibiting significant clonal expansion showed no link between *pvcrt*-o polymorphisms and reduced efficacy of chloroquine [57,58].

Amodiaquine and its clinically active metabolite, desethylamodiaquine, are structurally related to chloroquine, and polymorphisms of *pf*MRP1 are associated with an increased IC_50_ for both drugs in 15 adapted parasites [63]. Furthermore, evidence indicates chloroquine cross-resistance in parasites with mutations in *pfcrt* and *pfmdr1*. As a result, amodiaquine is used as a prophylactic treatment in combination with sulfadoxine–pyrimethamine and artesunate. Piperaquine combined with dihydroartemisinin has shown excellent efficacy and safety in treating falciparum malaria, but resistance has emerged in Cambodia, Ethiopia, Turkey, and Myanmar [78,79].

Reduced sensitivity of *P. falciparum* to quinine has been extensively reported in Asia and South America, but it appears to be relatively uncommon in Africa, where results regarding resistance have been inconsistent [59,60]. The resistance mechanism is complex and involves cross-resistance with other aryl amino alcohols and 4-aminoquinolines, suggesting a common genetic mechanism. Mutations in *pfmdr1* (N86Y and N1042D) and *pfcrt* (K76T and N75E or N326D) contribute to reduced susceptibility to quinine. *pfcrt* typically transports peptides originating from hemoglobin via the DV into the cytosol of parasites. However, *pfcrt* resistance mutations enable protein products for the transportation of these active forms from the digestive vacuole of the parasite again into the cytosol of parasites, preventing their accumulation in the vacuole.

Resistance to mefloquine in both *P. falciparum* and *P. vivax* has been thought to be fundamentally caused by an increased *mdr1* copy number, unlike chloroquine and antifolate drugs, where resistance is due to point mutations [56]. However, there is evidence of amino acid substitutions in *pvmdr1* [80]. Resistance to primaquine, seen in *P. vivax*, is difficult to determine due to reinfections in endemic areas, although whole-genome sequencing has identified polymorphisms in potential resistance genes [81]. Despite their effectiveness, 8-aminoquinoline compounds such as primaquine have several limitations and associated resistance issues. One major limitation is their potential for causing hemolysis in patients with a deficiency in glucose-6-phosphate dehydrogenase (G6PD), a genetic condition that affects a significant proportion of populations in malaria-endemic regions [82]. G6PD deficiency affects red blood cells’ ability to handle the oxidative stress caused by these drugs. Primaquine, in particular, can cause hemolysis in individuals with a G6PD deficiency. Before administering primaquine or tafenoquine, patients must be screened for G6PD deficiency to avoid severe hemolytic reactions, which can be life threatening.

Antifolate resistance develops due to single-point gene mutations encoding enzymes such as dihydrofolate reductase (DHFR) and dihydropteroate synthase (DHPS). These point mutations (N51I, C59R, S108N, and I164L) in parasite *dhfr* affect pyrimethamine binding to the dhfr active site, decreasing enzyme activity. This results in adverse fitness effects on the parasite [83,84]. Despite a decline in *dhfr* mutants in areas where first-line treatments changed to ACTs, they remain prevalent in regions using SP in combination therapies or for intermittent preventive therapy. The persistence of these mutations may also be linked to the use of trimethoprim–sulfamethoxazole in HIV-positive individuals. Increasing the gene dosage may play a compensatory role in antifolate resistance by boosting the pathway’s flux to offset the reduced efficacy of DHFR and/or DHPS genes carrying resistance mutations [85]. Genome scanning identified an amplification around GTP-cyclohydrolase 1 (*gch1*), which lessens the influence of downstream drug resistance mutations in the folate synthesis pathway [86].

Resistance to artemisinin by *P. falciparum* has been detected mainly in Southeast Asia [87]. These resistant strains have the potential to spread in various global regions and could become a global threat for malaria control and treatment. Artemisinin resistance is mainly driven by mutation in *kelch13* (*k13*) likely as a secondary effect of mutations in several genes of *P. falciparum*, including *pfmdr1*, *pfatp6*, *pfmdr2*, or *pfcrt*. Genetic mutation in such genes is linked with late parasitic clearance. Genome-wide analyses confirmed that late clearance of Cambodian parasites was strongly linked to *kelch13* and revealed additional genetic elements, for example, *pfcrt*, related to an extended duration of parasitic clearance [88]. Artemisinin resistance to *P. vivax* remains relatively low, as no significant mutations in the *P. falciparum pfk13* gene have been reported. However, the presence of polymorphisms in other genes, such as *pvmdr1* and *pvdhfr,* indicates evolving drug resistance patterns, underscoring the need for ongoing monitoring and the adaptation of treatment strategies to maintain the efficacy of current therapies, like DHA–PPQ [89].

Resistance to lumefantrine has not been clearly demonstrated in clinical isolates, but *pfmdr1* gene amplification in *P. falciparum* and *P. vivax* is linked to a higher risk of treatment failure with artemether–lumefantrine (Coartem^®^) [90]. Furthermore, findings suggested that using lumefantrine and mefloquine in ACTs may promote the selection of *pfmdr1*, *pfcrt*, and *pfk13* polymorphisms, which may drive resistance [91]. This may be due to the gene product influencing the access and/or effectiveness of these aryl amino alcohol drugs at sites of hemoglobin digestion and heme metabolism.

## 4. Innovative Approaches to the Treatment of Resistance

To prevent drug resistance, malaria treatments are administered using a combination of different drugs and varying interventions. This approach targets various phases in the lifecycle of malaria parasite, reducing the selective pressure on susceptible genes to antimalarial therapy. Unfortunately, as discussed above, there are reports of total or partial resistance to different dual antimalarial therapies.

### 4.1. Triple Artemisinin-Based Combination Therapies (TACTs)

The short half-life of artemisinin in ACTs can result in parasites being exposed to the partner drug alone, increasing resistance risk. Recently, the use of triple artemisinin-containing combination therapies (TACTs) has been debated. TACTs would include an artemisinin component plus two current ACT partner drugs, with combinations like piperaquine, mefloquine, lumefantrine, and amodiaquine being frequently proposed due to their antagonistic resistance mechanisms. Other combinations being tested include an ACT with atovaquone–proguanil.

In a recent large-scale trial involving 97.000 subjects, artemisinin–piperaquine (AP) with or without low-dose primaquine (LDPMQ) was administered in three monthly rounds across Anjouan Island, the Union of the Comoros [92]. The study evaluated *P. falciparum* malaria rates, mortality, parasitemia, adverse events, and PfK13 Kelch-propeller gene polymorphisms. The administration of artemisinin–piperaquine (AP) with or without LDPMQ significantly reduced the malaria rates on Anjouan Island, the Union of the Comoros. Coverage was high, with 85–93% of the population receiving treatment. Monthly malaria hospital rates dropped dramatically in both the AP + LDPMQ and AP-only groups, with reductions from 310.8 to 2.06 per 100,000 people and from 412.1 to 2.60, respectively. Both regimens showed high effectiveness (AP + LDPMQ: 99.08%; AP alone: 99.13%) and were well tolerated without severe adverse events. Additionally, there was no evidence of selection for PfK13 Kelch-propeller mutations in the malaria samples analyzed post-MDA.

Another recent large-scale trial evaluated several antimalarial treatments: DHA-PPQ, artesunate–mefloquine (AS-MQ), DHA-PPQ plus mefloquine, artemether–lumefantrine (AT-L), and AT-L plus amodiaquine [93]. In regions of Cambodia, Thailand, and Vietnam with piperaquine resistance, the research study found that DHA-PPQ plus mefloquine had a significantly higher 42-day PCR-corrected efficacy (98%) compared to DHA-PPQ alone (48%). In Myanmar, DHA-PPQ plus mefloquine had a 91% efficacy, while dihydroartemisinin–piperaquine alone had a 100% efficacy. The efficacy of artesunate–mefloquine in Cambodia was similar to that of DHA-PPQ plus mefloquine (95% vs. 96%). The efficacy of artemether–lumefantrine plus amodiaquine was comparable to artemether–lumefantrine alone (98% vs. 97%). Early vomiting was more frequent with DHA-PPQ plus mefloquine compared to DHA-PPQ alone, but vomiting was infrequent for both AT-L combinations. Adding amodiaquine to AT-L increased the QT interval, while adding mefloquine to DHA-PPQ did not induce such an effect.

Piperaquine resistance is widespread, and parasites resistant to both mefloquine and piperaquine have been identified, posing a significant risk of these resistant strains spreading [94]. The primary objective of using DHA-PPQ plus mefloquine is not solely to offer an effective treatment, since AS-MQ and artesunate–pyronaridine are still highly effective. Instead, the goal is to delay the re-emergence of mefloquine resistance and to ensure effective treatment when it does occur [95]. However, given the widespread resistance to piperaquine and the identification of parasites resistant to both mefloquine and piperaquine, there is a significant risk of these resistant parasites spreading. This might explain why combining mefloquine with DHA-PPQ in regions where parasites are resistant to piperaquine but sensitive to mefloquine leads to high efficacy in the GMS. As such, switching to TACTs post-development of resistance to the component drugs may limit the potential benefits for resistance prevention. Moreover, while studies have reported TACTs to be safe and well tolerated, introducing another drug to existing regimens necessitates further research on tolerability, toxicity, and drug interactions.

In Africa, AT-L and artesunate–amodiaquine (AS-AQ) are effective treatments. The goal of combining these drugs in a TACT or prolonging the treatment duration is to mitigate the development and transmission of lumefantrine and amodiaquine rather than to enhance patient outcomes [96]. As the most widely recommended first-line treatments, the loss of AT-L and AS-AQ before new therapies become available would be catastrophic. The potential delay in resistance benefiting future patients must be balanced against the increased risk of drug interactions and the resources required to develop and implement TACTs or extended treatment regimens. Using co-blistered treatments, which involve packaging individual drugs together, rather than co-formulated ones, could result in the misuse of individual drugs. Emphasizing the optimal use of existing treatments, advancing the development of new antimalarials, and concentrating on other interventions such as vector control may yield greater benefits for at-risk populations.

### 4.2. Antimalarial Vaccine Development

Malaria is an ongoing global health issue, especially in non-Mediterranean Africa, where the majority of malaria-related health complications and fatalities occur [97]. While chemotherapeutic interventions and vector control strategies have historically been the cornerstones of malaria management, the onset of resistance to antimalarial drugs and the logistical challenges of sustaining vector control efforts have underscored the demand for additional preventive measures. Antimalarial vaccines represent a promising adjunct to existing malaria control strategies, offering the potential for long-term protection against infection and transmission.

The quest for a malaria vaccine has been a long and arduous journey, marked by numerous scientific and logistical challenges. Early efforts in the 20th century focused on whole parasite vaccines, which faced significant hurdles due to the complexity of the Plasmodium life cycle and the parasite’s ability to evade the immune system [98]. The advent of molecular biology and advances in immunology have since paved the way for more targeted approaches, leading to the development of subunit vaccines and, more recently, genetically attenuated parasites and vectored vaccines [99].

#### 4.2.1. Preclinical and Clinical Studies

##### Pre-Erythrocytic-Stage Vaccines

These vaccines target the sporozoite and liver stages of the Plasmodium parasite, aiming to prevent the parasite from reaching the blood stage, where it causes symptomatic illness.

##### Whole Sporozoite Vaccines (WSVs)

a. Radiation-attenuated sporozoites (RASs): Radiation-attenuated sporozoite (RAS) vaccines represent a promising approach to malaria prevention by targeting the early stages of the Plasmodium parasite’s lifecycle. These vaccines use sporozoites that have been weakened through controlled exposure to gamma radiation, allowing them to invade liver cells but preventing them from maturing to the blood stage, which causes the symptomatic phase of malaria [100]. This attenuation process damages the DNA of the sporozoites, rendering them incapable of further development once they infect liver cells, thereby stimulating the host’s immune system without causing disease [101].

A prime example of an RAS vaccine is the PfSPZ vaccine developed by Sanaria. This vaccine uses attenuated sporozoites of *Plasmodium falciparum*, the most lethal malaria parasite species. Clinical trials have shown promising results; in one notable study, over 90% of participants who received the vaccine were protected against malaria, indicating a high efficacy rate [102]. Such findings suggest that RAS vaccines could significantly reduce malaria transmission if widely deployed.

The advantages of RAS vaccines include the broad antigen presentation they offer compared to subunit vaccines that target a single antigen. This broad exposure potentially provides more robust and comprehensive protection [103]. Additionally, the immune response induced by RAS vaccines tends to be long-lasting, reducing the need for frequent booster doses [104].

However, there are challenges associated with RAS vaccines. Producing large quantities of attenuated sporozoites is technically challenging and costly, involving the raising of mosquitoes, infecting them with the malaria parasite, and then irradiating the sporozoites harvested from the mosquitoes [105]. Furthermore, the PfSPZ vaccine requires intravenous injection, posing logistical challenges, especially in resource-limited settings, as this route ensures a sufficient number of sporozoites reach the liver to induce a protective immune response [106].

Future directions in the development of RAS vaccines include optimizing the production and delivery processes, such as improving the sporozoite production efficiency and developing formulations for intramuscular or subcutaneous administration [107]. Researchers are also exploring combination strategies, integrating RAS vaccines with other malaria interventions like chemoprophylaxis or different vaccine platforms to enhance their overall efficacy and provide broader protection [108].

b. Genetically attenuated parasite (GAP) vaccines are a novel approach in the fight against malaria. These vaccines use genetically modified sporozoites that are designed for inhibition during liver phase of the Plasmodium parasite’s lifecycle, preventing them from progressing to the blood phase and causing symptomatic disease. One prominent example of a GAP vaccine currently in clinical development is GAP3KO by Sanaria. This vaccine involves the deletion of three key genes in the *Plasmodium falciparum* genome, rendering the parasite incapable of completing its lifecycle in the human host.

The GAP approach offers several advantages over other vaccine strategies. By arresting the parasite in the liver stage, GAP vaccines aim to induce a strong and protective immune response. The modified sporozoites are able to infect liver cells, where they present a broad range of antigens to the immune system, eliciting robust CD8+ and CD4+ T-cell responses. These immune responses are crucial for eliminating the parasite during its hepatic stage and preventing its entry into the bloodstream [109].

Clinical trials of GAP vaccines, such as GAP3KO, have shown promising results. Early studies have demonstrated the vaccine tolerability and immunogenic response, with participants developing strong cellular and humoral immunity. The ability of GAP vaccines to provide sterilizing immunity—completely preventing infection—makes them a highly attractive option for malaria control and eradication efforts [107].

However, there are challenges associated with the development and deployment of GAP vaccines. The genetic modifications required to create attenuated parasites must be precise and ensure that the parasite is completely safe for human use. Additionally, the production of genetically modified sporozoites on a large scale remains a complex and costly process. Overcoming these challenges is essential to making GAP vaccines a viable tool to achieve the goal to combat malaria worldwide [108].

##### Subunit Vaccines

Antimalarial subunit vaccines are a promising approach to malaria prevention that focus on targeting specific proteins or antigens from the malaria parasite, Plasmodium spp. Unlike whole-organism vaccines, which use weakened or inactivated forms of the parasite, subunit vaccines use only a part of the parasite’s structure. These components, often recombinant proteins, are selected for their ability to provoke a strong immune response that can prevent infection or reduce the severity of the disease [98,110].

a. RTS,S/AS01: One of the most significant milestones in malaria vaccine development is the vaccine RTS,S/AS01 (Mosquirix) which gathered favorable scientific views from the European Medicines Agency in 2015 and has since been piloted in several African countries [111]. RTS,S/AS01, a bioengineered protein vaccine composed of (CSP) from *P. falciparum* and a hepatitis B surface antigen (HBsAg), formulated using the AS01 adjuvant system to enhance it [111].

The RTS,S/AS01 (Mosquirix) vaccine underwent a Phase 3 trial from 2009 to 2014 across seven sub-Saharan African countries [112]. The study involved 15,459 participants comprising 6537 infants (6–12 weeks) and 8922 children (5–17 months). The participants were allocated randomly to undergo three RTS,S/AS01 doses after 0, 1, and 2 months, which was followed by a booster after 20 months, or control vaccines. The trial was double -blind and completed in two phases: a double-blind phase (0–32 months) and an extension phase (33 months until closure). Follow-up lasted a median of 38 months for infants and 48 months for older children. The primary aim was to assess the vaccine’s efficacy against clinical malaria over 12 months post-vaccination. The data showed an efficacy of 31.3% in infants and 55.8% in children aged between 5 and 17 months. Over the entire follow-up, the four-dose efficacy was 25.9% in infants and 36.3% in older children. However, the efficacy declined over time, but more slowly in the four-dose group. The vaccine showed lower efficacy in infants compared to older children. Severe malaria was rare, likely due to prompt treatment for clinical malaria in the study participants. While not a panacea, RTS,S is a proof of concept that a malaria vaccine is feasible and provides valuable insights for the development of next-generation vaccines.

b. PfCSP (*Plasmodium falciparum* Circumsporozoite Protein) vaccines:

The CSP of *P. falciparum* is a major malarial target for vaccines due to its essential function in the parasite’s lifecycle. CSP is present on the surface of sporozoites, the form of the parasite that infects the liver, making it a prime candidate for vaccine development [113]. PfCSP is another subunit vaccine that has been in preclinical studies. One of the leading PfCSP-based vaccine candidates is R21/Matrix-M, which was developed by the University of Oxford in collaboration with Novavax. This vaccine has shown high efficacy in early trials, marking a significant advancement in the fight against malaria.

R21/Matrix-M consists of the R21 antigen, which is a virus-like particle expressing the CSP, and the Matrix-M adjuvant, which enhances the immune response [114]. The adjuvant is crucial in increasing the magnitude and durability of the immune response, making the vaccine more effective. In a Phase IIb trial conducted in Burkina Faso, R21/Matrix-M demonstrated a vaccine efficacy of 77% over 12 months child with age between 5 and 17 months old, who were vaccinated with three doses followed by a booster shot [115]. This is a significant improvement over RTS,S/AS01, the initial vaccine for malaria to receive regulatory approval. The high efficacy observed in this trial has generated optimism about the potential of R21/Matrix-M to significantly reduce malaria morbidity and mortality if similar results are achieved in larger Phase III trials [115]. The safety profile of R21/Matrix-M has been favorable, with most adverse events being mild to moderate in severity. The inclusion of the Matrix-M adjuvant has not resulted in any significant increase in adverse events compared to other vaccines [115,116].

Despite the promising results, there are several challenges associated with CSP-based vaccines. Firstly, the genetic diversity of the CSP in different P. falciparum strains can affect vaccine efficacy. Efforts are being made to include multiple epitopes in vaccine formulations to overcome this issue [117]. Secondly, the durability of the immune response is a critical factor. Booster doses, as seen with R21/Matrix-M, may be necessary to maintain high levels of protection over time [116].

##### Blood-Stage Vaccines

Targeting the merozoite stage of the parasite, these vaccines aim to reduce the parasite burden in the blood and alleviate the symptoms of malaria. Candidates include vaccines based on merozoite surface proteins (MSPs) and other erythrocyte invasion ligands.

**Figure 1:**
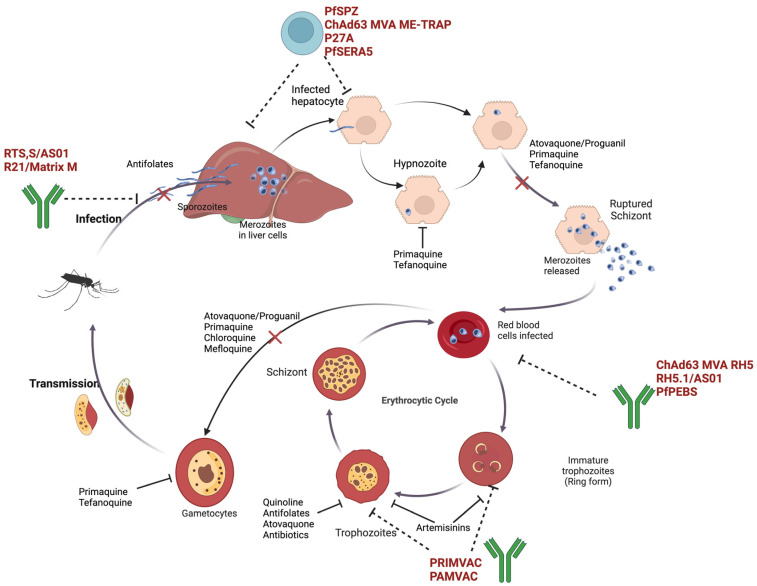
Interventions in life cycle of parasitic malaria: drug and vaccine targets.

The asexual stages of Plasmodium, involving repeated replication cycles within erythrocytes, are prime targets for malaria vaccine development. Strain-specific immunity due to antigen polymorphism has traditionally hindered vaccine efficacy. Hence, utilizing conserved antigens that are naturally acquired shows potential for higher efficacy [118]. The *Plasmodium falciparum* Reticulocyte-binding Protein Homolog 5 (PfRipr) is a novel candidate for targeting the asexual blood stage by eliciting potent growth inhibitory antibodies against the parasite in red blood cells [119]. The PfRipr/PfCyRPA/Rh5 complex, particularly when combined with adjuvants, is promising for vaccine development. Notably, vaccines such as Rh5.1/AS01 and ChAd63.MVARh5, which induce the production of neutralizing antibodies, are in Phase 2 clinical trials (Figure 1). Additionally, developmental efforts targeting VAR2CSA, a variant surface antigen expressed by the *P. falciparum*, including PRIMVAC and PAMVAC, focus on efficacy against malaria during pregnancy [120].

During the liver stage, vaccines targeting the asexual stage induce IFN-γ synthesis by activating CD8^+^ T cells, leading to the production of antiparasitic nitric oxide (NO) by infected hepatocytes. Other mechanisms include apoptosis of infected hepatocytes and the recognition of parasite antigens by natural killer (NK) cells [121]. LSA-1-containing vaccines are potential candidates for clinical trials due to their specific expression by the parasite [120].

Merozoites, once they burst from infected cells, are vulnerable to circulating antibodies, making them a target for anti-merozoite vaccines (Figure 1). Mechanisms include blocking attachment sites, invasion, or development inside RBCs. Proteins such as the Duffy-binding protein (DBP) and erythrocyte-binding antigen (EBA-175) serve as receptors in RBCs for *P. vivax* and *P. falciparum*, respectively [122,123]. Blocking these receptors can prevent parasite invasion. Antibodies against the merozoite surface protein (MSP-1) have shown inhibitory effects on the growth of plasmodium both in vitro and in vivo [124]. Blood-stage vaccines can reduce clinical illness, induce sterile immunity, and decrease transmission by controlling parasite density and reducing gametocytes in the bloodstream [125].

Infected RBCs express parasitic antigens, and asexual-stage vaccines act on these cells through cytokine release by CD4+ T cells, inducing parasiticidal and parasitostatic effects. This activation leads to macrophage activation and antibody-mediated complement system activation or opsonization. Antibodies against parasitized RBCs facilitate phagocytosis, preventing cerebral malaria. Ring erythrocyte surface antigen (RESA) combined with MSP-1 and 2 has been tested in clinical trials [126]. Tumor necrosis factor-alpha (TNF-α) is a significant focus of vaccine innovation against parasite toxins.

When sporozoites are transmitted through a mosquito bite, they travel to the liver, infecting the organ. Here, vaccines like RTS,S/AS01 and R21/Matrix M elicit an immune response through antibody generation that prevents hepatocyte infection. In the liver, sporozoites develop into schizonts, with some becoming dormant hypnozoites, which be reactivated in the future, causing disease relapse. Vaccines like PfSPZ and P27A in in different stages of clinical trials, and they induce the activation of T cells that directly attack infected hepatocytes. Drugs like atovaquone–proguanil and primaquine also target this stage to prevent relapse. In the blood stage, merozoites infect red blood cells. Vaccines like RH5.1/AS01 and PRIMVAC PfEBS and drugs (e.g., quinolines, antifolates, and artemisinins) target different stages of the erythrocytic cycle to kill the parasite. During the transmission stage, gametocytes develop and are transmitted to mosquitoes to cause re-infection. Drugs like primaquine aim to block transmission by targeting these gametocytes.

## 5. Challenges and Future Directions

One of the most pressing challenges in malaria therapy is the development of drug resistance, particularly against ACTs, which are the frontline treatment for *Plasmodium falciparum* malaria [9]. Resistance has been documented in Southeast Asia and poses a significant threat to global malaria control efforts [40]. The widespread artemisinin resistance might bring about increased malaria morbidity and mortality, as fewer effective treatment options would remain available. Another major challenge is the lack of highly effective vaccines. While some vaccines, such as RTS,S/AS01 (Mosquirix), have been approved, they offer only moderate protection and require multiple doses [111]. The efficacy of RTS,S wanes over time, and its ability to reduce severe malaria and mortality is limited. Therefore, developing more effective vaccines that provide long-lasting immunity and can be widely deployed in endemic areas is crucial.

The complex life cycle of the Plasmodium parasite, involving both human and mosquito hosts, further complicates treatment and prevention efforts [127]. The parasite undergoes several developmental stages, each with unique biological characteristics, making it difficult to target at multiple points in its life cycle. Additionally, the antigenic diversity and immune evasion strategies of Plasmodium make it challenging to develop therapies that are universally effective [128]. Compounding these biological challenges is the issue of poor health infrastructure in many malaria-endemic regions, particularly in sub-Saharan Africa and Southeast Asia [129]. Inadequate health infrastructure makes it difficult to deliver effective malaria treatment and prevention measures, with limited access to healthcare, a lack of trained medical personnel, and insufficient supply chains for drugs and diagnostics posing significant barriers to malaria control efforts.

Financial and logistical barriers also hinder progress in malaria therapy. The high cost of developing new antimalarial drugs and vaccines, coupled with the logistical challenges of conducting clinical trials in endemic regions, impedes progress. Sustained financial investment is required for ongoing research, development, and deployment of new interventions, which is difficult to maintain in resource-limited settings [130]. Furthermore, political and social instability in many malaria-endemic regions disrupts malaria control programs. Armed conflict, social unrest, and political instability can displace populations, making it difficult to reach affected communities with essential services such as bed nets, insecticides, and treatment [131]. Additionally, these issues can interrupt supply chains for antimalarial drugs, exacerbating the problem of drug resistance.

Emerging threats such as climate change, urbanization, and changes in land use patterns are altering the distribution and behavior of malaria vectors, potentially leading to the emergence of malaria in new areas [132]. These environmental changes could complicate existing malaria control efforts and necessitate new strategies to address shifting epidemiological patterns.

To address these challenges, the future of malaria therapy must focus on several key areas. Developing new antimalarial medicines with unique modes of action is critical to combat drug resistance. Research is ongoing to detect lead molecules that could overcome resistance and target different phases in the life cycle of parasites [133,134]. Combination therapies that include new drugs may be essential in hindering the onset of resistance. Next-generation vaccines that improve efficacy, durability, and coverage are also needed. Strategies under investigation include multi-antigen vaccines that target various stages in life cycle of parasites and vaccines that elicit stronger cellular and humoral immune responses [135,136]. Promising candidates like R21/Matrix-M are actively being tested in clinical trials and may offer higher efficacy compared to existing vaccines.

Advances in gene-editing technologies, such as CRISPR-Cas9, offer new possibilities for malaria therapy. These technologies are currently been employed to modify the mosquito vector, creating a species that is either resistant to Plasmodium infection, or to create genetically attenuated parasites for use in live-attenuated vaccines [137]. Gene drives, which spread specific genetic traits rapidly through mosquito populations, are also being explored as a way to reduce malaria transmission by hindering progression through the parasite life cycle [138]. Improving diagnostics and surveillance is another crucial area for future malaria therapy. Accurate and timely diagnosis is essential for effective malaria treatment and control. Future efforts should focus on developing more sensitive and specific diagnostic tools that can detect low-density infections and differentiate between species of Plasmodium. Enhanced surveillance systems, including mobile technology and data analytics, will improve tracking malaria cases and the effectiveness of interventions.

The application of personalized medicine in malaria therapy is an emerging area of interest, involving tailoring treatment based on the genetic makeup of the patient and the parasite. This approach could optimize drug efficacy, reduce adverse effects, and help manage drug resistance by identifying the most effective treatment regimens for individual patients. Sustained malaria control requires active participation from local communities. Future efforts should focus on empowering communities through education about malaria prevention and treatment, as well as involving them in the design and implementation of control programs. This grassroots approach can enhance the effectiveness and sustainability of interventions.

Finally, continued global collaboration and investment are crucial for advancing malaria therapy. Partnerships between governments, international organizations, academic institutions, and the private sector are essential. Increased funding for research and development, as well as for the deployment of new interventions, will be necessary to achieve the goal of malaria eradication. By addressing these challenges and pursuing these future directions, significant progress can be made in the fight against malaria, ultimately reducing the burden of this devastating disease.

## Figures and Tables

**Table 1:** Antimalarials, mechanism of actions, and genetic basis of resistance mechanism.

Name		Parasite Target	Mechanism of Action	Dosage	Side Effects	Resistance	Genetics of Resistance	Ref.
Quinine derivatives								
Quinine	* 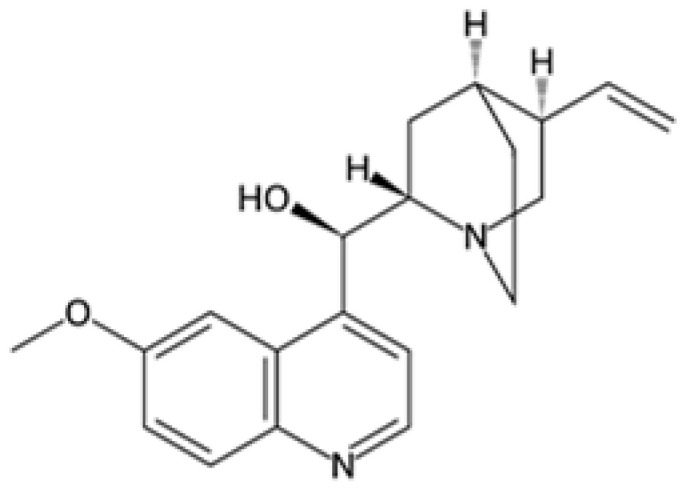 *	*P. falciparum*, *P. vivax*	Interferes with parasite’s ability to digest hemoglobin	600 mg 3 times daily for 7 days	Tinnitus, nausea, headache, and blurred vision	Resistance present in some regions	Mutations in Pfcrt (*P. falciparum* chloroquine resistance transporter) and Pfmdr1 (multidrug resistance gene 1)	[59,60,61]
Quinidine	* 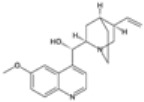 *	*P. falciparum*	Similar to quinine, blocks DNA replication	10 mg/kg loading dose, then 0.02 mg/kg/min	Arrhythmia, hypotension, and dizziness	Resistance reported	Similar resistance mechanisms as quinine, Pfcrt and Pfmdr1 mutations	[59,60,62]
Chloroquine	* 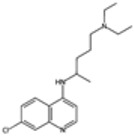 *	*P. falciparum*, *P. vivax*	Inhibits heme polymerase activity	25 mg/kg over 3 days	Itching, gastrointestinal upset, and retinopathy	Widespread resistance, especially in *P. falciparum*	Pfcrt mutations (particularly K76T) and Pfmdr1 mutations	[57]
Amodiaquine	* 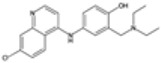 *	*P. falciparum*	Similar to chloroquine, disrupts heme digestion	10 mg/kg for 3 days	Agranulocytosis and hepatotoxicity	Resistance reported	Pfcrt and Pfmdr1 mutations	[63,64]
Mefloquine	* 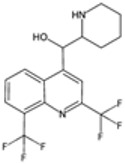 *	*P. falciparum*	Inhibits heme polymerization	250 mg weekly for prophylaxis	Neuropsychiatric effects and gastrointestinal upset	Resistance in Southeast Asia	Amplification and mutations in Pfmdr1	[56,65]
Halofantrine	* 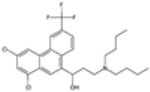 *	*P. falciparum*	Interferes with heme metabolism	8 mg/kg body weight, then repeat in 6 h	Cardiotoxicity and gastrointestinal upset	Limited use due to resistance	Pfmdr1 mutations	[13,66]
Piperaquine	* 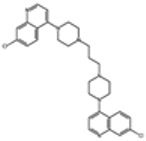 *	*P. falciparum*	Similar to chloroquine, disrupts heme digestion	160–1600 mg daily for 3 days	QT prolongation and gastrointestinal upset	Emerging resistance	Amplification and mutations in Pfmdr1 and Pfpm2 (plasmepsin 2)	[67,68]
Lumefantrine	* 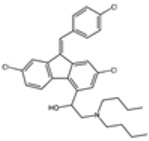 *	*P. falciparum*	Interferes with heme metabolism	480 mg twice daily for 3 days	Headache, dizziness, and gastrointestinal upset	Resistance emerging	Pfmdr1 mutations	[69,70]
8-Aminoquinoline								
Primaquine	* 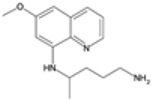 *	*P. vivax*, *P. ovale*	Generates reactive oxygen species and disrupts mitochondria	15 mg daily for 14 days	Hemolysis in G6PD-deficient patients and nausea	Some evidence of reduced efficacy	G6PD (glucose-6-phosphate dehydrogenase) deficiency affects drug efficacy and safety, no specific parasite gene mutations	[71,72]
Tafenoquine	* 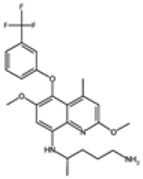 *	*P. vivax*	Similar to primaquine, disrupts mitochondria	100–300 mg	Hemolysis in G6PD-deficient patients and dizziness	Limited reports of resistance	G6PD deficiency impacts drug safety and efficacy, no known specific parasite resistance	[22,24,73]
Antifolate compounds								
Sulfadoxine	* 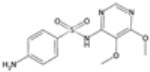 *	*P. falciparum*	Inhibits dihydropteroate synthase	500 mg as single dose	Rash, gastrointestinal upset, and Stevens–Johnson syndrome	Resistance common in many regions	Mutations in Pfdhps (*P. falciparum* dihydropteroate synthase), particularly A437G and K540E	[74]
Pyrimethamine	* 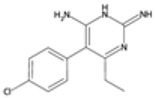 *	*P. falciparum*	Inhibits dihydrofolate reductase	25 mg single dose	Anemia, rash, and gastrointestinal upset	Resistance common in many regions	Mutations in Pfdhfr (*P. falciparum* dihydrofolate reductase), particularly N51I, C59R, S108N, and I164L	[28,74]
Proguanil	* 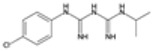 *	*P. falciparum*	Prodrug, inhibits dihydrofolate reductase	200 mg daily	Mouth ulcers and gastrointestinal upset	Some resistance reported	Pfdhfr mutations (similar to pyrimethamine)	[28,75]
Chlorproguanil	* 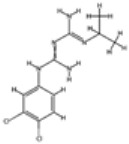 *	*P. falciparum*	Inhibits dihydrofolate reductase	20 mg daily	Hemolysis in G6PD-deficient patients and nausea	Withdrawn due to safety concerns	Pfdhfr mutations (similar to pyrimethamine and proguanil)	[76]
Artemisinin compounds								
Artemisinin	* 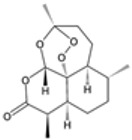 *	*P. falciparum*	Generates reactive oxygen species and damages proteins and membranes	250–1000 mg daily for 7 days	Nausea, dizziness, and neutropenia	Resistance emerging in Southeast Asia	Mutations in Pfk13 (Kelch 13 gene), particularly C580Y, R539T, and Y493H	[10,77]
Artesunate	* 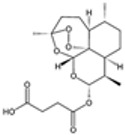 *	*P. falciparum*	Similar to artemisinin, more water soluble	200 mg daily for 7 days	Hemolysis and gastrointestinal upset	Resistance emerging in Southeast Asia	Pfk13 mutations (similar to artemisinin)	[43,44,51]
Artemether	* 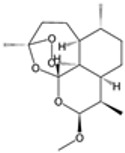 *	*P. falciparum*	Similar to artemisinin, lipid-soluble	160 mg daily for 3 days	Fever, nausea, and headache	Resistance emerging in Southeast Asia	Pfk13 mutations (similar to artemisinin)	[16,30,43]
Dihydroartemisinin	* 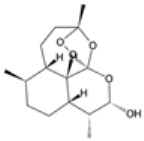 *	*P. falciparum*	Active metabolite of artemisinin, generates reactive oxygen species	200 mg daily for 7 days	Nausea, dizziness, and anemia	Resistance emerging in Southeast Asia	Pfk13 mutations (similar to artemisinin)	[28,45,46]

## References

[1] Al-Awadhi M., Ahmad S., Iqbal J. (2021). Current Status and the Epidemiology of Malaria in the Middle East Region and Beyond. Microorganisms.

[2] Aliyo A., Golicha W., Fikrie A. (2024). Malaria and associated factors among under-five children in Borena pastoral communities, southern Ethiopia. Front. Parasitol..

[3] Anwar M., Tarique M. (2024). Introduction: An Overview of Malaria and Plasmodium. Drug Targets for Plasmodium falciparum: Historic to Future Perspectives.

[4] Zekar L., Sharman T. (2024). Plasmodium falciparum Malaria. StatPearls.

[5] Varo R., Chaccour C., Bassat Q. (2020). Update on malaria. Med. Clínica Engl. Ed..

[6] Kotepui M., Kotepui K.U., Milanez G.D.J., Masangkay F.R. (2020). Prevalence and risk factors related to poor outcome of patients with severe Plasmodium vivax infection: A systematic review, meta-analysis, and analysis of case reports. BMC Infect. Dis..

[7] Naik D.G. (2020). Plasmodium knowlesi-mediated zoonotic malaria: A challenge for elimination. Trop. Parasitol..

[8] Oriero E.C., Amenga-Etego L., Ishengoma D.S., Amambua-Ngwa A. (2021). Plasmodium malariae, current knowledge and future research opportunities on a neglected malaria parasite species. Crit. Rev. Microbiol..

[9] Dagen M., Patrick G.L. (2020). Chapter 1—History of malaria and its treatment. Antimalarial Agents.

[10] Dhorda M., Amaratunga C., Dondorp A.M. (2021). Artemisinin and multidrug-resistant *Plasmodium falciparum*—A threat for malaria control and elimination. Curr. Opin. Infect. Dis..

[11] Siqueira-Neto J.L., Wicht K.J., Chibale K., Burrows J.N., Fidock D.A., Winzeler E.A. (2023). Antimalarial drug discovery: Progress and approaches. Nat. Rev. Drug Discov..

[12] Pandey S.K., Anand U., Siddiqui W.A., Tripathi R. (2023). Drug Development Strategies for Malaria: With the Hope for New Antimalarial Drug Discovery—An Update. Adv. Med..

[13] Shibeshi M.A., Kifle Z.D., Atnafie S.A. (2020). Antimalarial Drug Resistance and Novel Targets for Antimalarial Drug Discovery. Infect. Drug Resist..

[14] Kapishnikov S., Hempelmann E., Elbaum M., Als-Nielsen J., Leiserowitz L. (2021). Malaria Pigment Crystals: The Achilles′ Heel of the Malaria Parasite. ChemMedChem.

[15] Biswas P., Roy R., Ghosh K., Nath D., Samadder A., Nandi S. (2024). To quest new targets of Plasmodium parasite and their potential inhibitors to combat antimalarial drug resistance. J. Parasit. Dis..

[16] Wilkins C.A., du Plessis L.H., Viljoen J.M. (2021). Investigating In Vitro and Ex Vivo Properties of Artemether/Lumefantrine Double-Fixed Dose Combination Lipid Matrix Tablets Prepared by Hot Fusion. Pharmaceutics.

[17] Popovici J., Tebben K., Witkowski B., Serre D. (2021). Primaquine for Plasmodium vivax radical cure: What we do not know and why it matters. Int. J. Parasitol. Drugs Drug Resist..

[18] Capela R., Moreira R., Lopes F. (2019). An Overview of Drug Resistance in Protozoal Diseases. Int. J. Mol. Sci..

[19] Yadessa A.M., Zeleke D. (2021). A Review on Synthesis of Quinoline Analogs as Antimalarial, Antibacterial and Anticancer agents. Ethiop. J. Sci. Sustain. Dev..

[20] Chamma-Siqueira N.N., Negreiros S.C., Ballard S.-B., Farias S., Silva S.P., Chenet S.M., Santos E.J.M., de Sena L.W.P., da Costa F.P., Cardoso-Mello A.G.N. (2022). Higher-Dose Primaquine to Prevent Relapse of Plasmodium vivax Malaria. N. Engl. J. Med..

[21] Hanboonkunupakarn B., White N.J. (2022). Advances and roadblocks in the treatment of malaria. Br. J. Clin. Pharmacol..

[22] Lacerda M.V.G., Llanos-Cuentas A., Krudsood S., Lon C., Saunders D.L., Mohammed R., Yilma D., Batista Pereira D., Espino F.E.J., Mia R.Z. (2019). Single-Dose Tafenoquine to Prevent Relapse of Plasmodium vivax Malaria. N. Engl. J. Med..

[23] Wångdahl A., Sondén K., Wyss K., Stenström C., Björklund D., Zhang J., Hervius Askling H., Carlander C., Hellgren U., Färnert A. (2022). Relapse of Plasmodium vivax and Plasmodium ovale Malaria With and Without Primaquine Treatment in a Nonendemic Area. Clin. Infect. Dis..

[24] Zottig V.E., Carr K.A., Clarke J.G., Shmuklarsky M.J., Kreishman-Deitrick M. (2020). Army Antimalarial Drug Development: An Advanced Development Case Study for Tafenoquine. Mil. Med..

[25] Pethrak C., Posayapisit N., Pengon J., Suwanakitti N., Saeung A., Shorum M., Aupalee K., Taai K., Yuthavong Y., Kamchonwongpaisan S. (2022). New Insights into Antimalarial Chemopreventive Activity of Antifolates. Antimicrob. Agents Chemother..

[26] Abugri J., Ansah F., Asante K.P., Opoku C.N., Amenga-Etego L.A., Awandare G.A. (2018). Prevalence of chloroquine and antifolate drug resistance alleles in *Plasmodium falciparum* clinical isolates from three areas in Ghana. AAS Open Res..

[27] Shamshad H., Bakri R., Mirza A.Z. (2022). Dihydrofolate reductase, thymidylate synthase, and serine hydroxy methyltransferase: Successful targets against some infectious diseases. Mol. Biol. Rep..

[28] Sehrawat R., Rathee P., Khatkar S., Akkol E., Khayatkashani M., Nabavi S.M., Khatkar A. (2024). Dihydrofolate Reductase (DHFR) Inhibitors: A Comprehensive Review. Curr. Med. Chem..

[29] Nzila A. (2006). The past, present and future of antifolates in the treatment of *Plasmodium falciparum* infection. J. Antimicrob. Chemother..

[30] Laminou I.M., Issa I., Adehossi E., Maman K., Jackou H., Coulibaly E., Tohon Z.B., Ahmed J., Sanoussi E., Koko D. (2024). Therapeutic efficacy and tolerability of artemether-lumefantrine for uncomplicated *Plasmodium falciparum* malaria in Niger, 2020. Malar. J..

[31] L’Huillier A.G., Lau R., Bitnun A., Boggild A.K. (2020). Atovaquone-proguanil treatment failure in a case of pediatric *Plasmodium falciparum* infection: Malabsorption and resistance. Travel Med. Infect. Dis..

[32] Kurup N., Rajnani N. (2022). Chronology of Drug Development for Malaria. Drug Development for Malaria.

[33] Fernandes V.d.S., da Rosa R., Zimmermann L.A., Rogério K.R., Kümmerle A.E., Bernardes L.S.C., Graebin C.S. (2022). Antiprotozoal agents: How have they changed over a decade?. Arch. Pharm..

[34] Mbeye N., ter Kuile F.O., Davies M.-A., Phiri K., Egger M., Wandeler G. (2014). Cotrimoxazole prophylactic treatment prevents malaria in children in sub-Saharan Africa: Systematic review and meta-analysis. Trop. Med. Int. Health TM IH.

[35] Egwu C.O., Augereau J.-M., Reybier K., Benoit-Vical F. (2021). Reactive Oxygen Species as the Brainbox in Malaria Treatment. Antioxidants.

[36] Egwu C.O., Tsamesidis I., Pério P., Augereau J.-M., Benoit-Vical F., Reybier K. (2021). Superoxide: A major role in the mechanism of action of essential antimalarial drugs. Free Radic. Biol. Med..

[37] Gupta Y., Goicoechea S., Pearce C.M., Mathur R., Romero J.G., Kwofie S.K., Weyenberg M.C., Daravath B., Sharma N., Poonam (2022). The emerging paradigm of calcium homeostasis as a new therapeutic target for protozoan parasites. Med. Res. Rev..

[38] Erhunse N., Sahal D. (2021). Protecting future antimalarials from the trap of resistance: Lessons from artemisinin-based combination therapy (ACT) failures. J. Pharm. Anal..

[39] Wicht K.J., Mok S., Fidock D.A. (2020). Molecular Mechanisms of Drug Resistance in *Plasmodium falciparum* Malaria. Annu. Rev. Microbiol..

[40] Derbie A., Mekonnen D., Adugna M., Yeshitela B., Woldeamanuel Y., Abebe T. (2020). Therapeutic Efficacy of Artemether-Lumefantrine (Coartem^®^) for the Treatment of Uncomplicated Falciparum Malaria in Africa: A Systematic Review. J. Parasitol. Res..

[41] Mumtaz R., Okell L.C., Challenger J.D. (2020). Asymptomatic recrudescence after artemether–lumefantrine treatment for uncomplicated falciparum malaria: A systematic review and meta-analysis. Malar. J..

[42] Maude R.J., Plewes K., Faiz M.A., Hanson J., Charunwatthana P., Lee S.J., Tärning J., Yunus E.B., Hoque M.G., Hasan M.U. (2009). Does Artesunate Prolong the Electrocardiograph QT Interval in Patients with Severe Malaria?. Am. J. Trop. Med. Hyg..

[43] Marwa K., Kapesa A., Baraka V., Konje E., Kidenya B., Mukonzo J., Kamugisha E., Swedberg G. (2022). Therapeutic efficacy of artemether-lumefantrine, artesunate-amodiaquine and dihydroartemisinin-piperaquine in the treatment of uncomplicated *Plasmodium falciparum* malaria in Sub-Saharan Africa: A systematic review and meta-analysis. PLoS ONE.

[44] Zwang J., Dorsey G., Mårtensson A., d’Alessandro U., Ndiaye J.-L., Karema C., Djimde A., Brasseur P., Sirima S.B., Olliaro P. (2014). *Plasmodium falciparum* clearance in clinical studies of artesunate-amodiaquine and comparator treatments in sub-Saharan Africa, 1999–2009. Malar. J..

[45] Mohammed H., Sime H., Hailgiorgis H., Gubae K., Haile M., Solomon H., Etana K., Girma S., Bekele W., Chernet M. (2022). Therapeutic efficacy of dihydroartemisinin–piperaquine for the treatment of uncomplicated Plasmodium vivax malaria in Seacha area, Arbaminch Zuria District, South West Ethiopia. Malar. J..

[46] Popovici J., Vantaux A., Primault L., Samreth R., Piv E.P., Bin S., Kim S., Lek D., Serre D., Menard D. (2018). Therapeutic and Transmission-Blocking Efficacy of Dihydroartemisinin/Piperaquine and Chloroquine against Plasmodium vivax Malaria, Cambodia. Emerg. Infect. Dis..

[47] Carrara V.I., Sirilak S., Thonglairuam J., Rojanawatsirivet C., Proux S., Gilbos V., Brockman A., Ashley E.A., McGready R., Krudsood S. (2006). Deployment of early diagnosis and mefloquine-artesunate treatment of falciparum malaria in Thailand: The Tak Malaria Initiative. PLoS Med..

[48] Nosten F., van Vugt M., Price R., Luxemburger C., Thway K.L., Brockman A., McGready R., ter Kuile F., Looareesuwan S., White N.J. (2000). Effects of artesunate-mefloquine combination on incidence of *Plasmodium falciparum* malaria and mefloquine resistance in western Thailand: A prospective study. Lancet Lond. Engl..

[49] Dondorp A.M., Nosten F., Yi P., Das D., Phyo A.P., Tarning J., Lwin K.M., Ariey F., Hanpithakpong W., Lee S.J. (2009). Artemisinin Resistance in *Plasmodium falciparum* Malaria. N. Engl. J. Med..

[50] Price R.N., Nosten F., Luxemburger C., Kham A., Brockman A., Chongsuphajaisiddhi T., White N.J. (1995). Artesunate versus artemether in combination with mefloquine for the treatment of multidrug-resistant falciparum malaria. Trans. R. Soc. Trop. Med. Hyg..

[51] Sirima S.B., Ogutu B., Lusingu J.P.A., Mtoro A., Mrango Z., Ouedraogo A., Yaro J.B., Onyango K.O., Gesase S., Mnkande E. (2016). Comparison of artesunate–mefloquine and artemether–lumefantrine fixed-dose combinations for treatment of uncomplicated *Plasmodium falciparum* malaria in children younger than 5 years in sub-Saharan Africa: A randomised, multicentre, phase 4 trial. Lancet Infect. Dis..

[52] Lee S.J., ter Kuile F.O., Price R.N., Luxemburger C., Nosten F. (2017). Adverse effects of mefloquine for the treatment of uncomplicated malaria in Thailand: A pooled analysis of 19,850 individual patients. PLoS ONE.

[53] Belete T.M. (2020). Recent Progress in the Development of New Antimalarial Drugs with Novel Targets. Drug Des. Devel. Ther..

[54] Ibraheem Z.O., Abd Majid R., Noor S.M., Sedik H.M., Basir R. (2014). Role of Different Pfcrt and Pfmdr-1 Mutations in Conferring Resistance to Antimalaria Drugs in *Plasmodium falciparum*. Malar. Res. Treat..

[55] Habtamu K., Petros B., Yan G. (2022). Plasmodium vivax: The potential obstacles it presents to malaria elimination and eradication. Trop. Dis. Travel Med. Vaccines.

[56] Nureye D., Salahaddin M., Zewudie A. (2020). Current Medicines for Malaria Including Resistance Issues. J. Pharmacol. Pharmacother..

[57] Rumaseb A., Moraes Barros R.R., Sá J.M., Juliano J.J., William T., Braima K.A., Barber B.E., Anstey N.M., Price R.N., Grigg M.J. (2023). No Association between the Plasmodium vivax crt-o MS334 or In9pvcrt Polymorphisms and Chloroquine Failure in a Pre-Elimination Clinical Cohort from Malaysia with a Large Clonal Expansion. Antimicrob. Agents Chemother..

[58] Orjuela-Sánchez P., de Santana Filho F.S., Machado-Lima A., Chehuan Y.F., Costa M.R.F., Alecrim M.d.G.C., del Portillo H.A. (2009). Analysis of Single-Nucleotide Polymorphisms in the crt-o and mdr1 Genes of Plasmodium vivax among Chloroquine-Resistant Isolates from the Brazilian Amazon Region. Antimicrob. Agents Chemother..

[59] Mayxay M., Barends M., Brockman A., Jaidee A., Nair S., Sudimack D., Pongvongsa T., Phompida S., Phetsouvanh R., Anderson T. (2007). In Vitro Antimalarial Drug Susceptibility and Pfcrt Mutation Among Fresh *Plasmodium falciparum* Isolates from the Lao PDR (Laos). Am. J. Trop. Med. Hyg..

[60] Legrand E., Volney B., Meynard J.-B., Mercereau-Puijalon O., Esterre P. (2008). In vitro monitoring of *Plasmodium falciparum* drug resistance in French Guiana: A synopsis of continuous assessment from 1994 to 2005. Antimicrob. Agents Chemother..

[61] Ogetii G.N., Akech S., Jemutai J., Boga M., Kivaya E., Fegan G., Maitland K. (2010). Hypoglycaemia in severe malaria, clinical associations and relationship to quinine dosage. BMC Infect. Dis..

[62] Wroblewski H.A., Kovacs R.J., Kingery J.R., Overholser B.R., Tisdale J.E. (2012). High Risk of QT Interval Prolongation and Torsades de Pointes Associated with Intravenous Quinidine Used for Treatment of Resistant Malaria or Babesiosis. Antimicrob. Agents Chemother..

[63] Henry M., Briolant S., Fontaine A., Mosnier J., Baret E., Amalvict R., Fusaï T., Fraisse L., Rogier C., Pradines B. (2008). In vitro activity of ferroquine is independent of polymorphisms in transport protein genes implicated in quinoline resistance in *Plasmodium falciparum*. Antimicrob. Agents Chemother..

[64] White N., Looareesuwan S., Edwards G., Phillips R., Karbwang J., Nicholl D., Bunch C., Warrell D. (1987). Pharmacokinetics of intravenous amodiaquine. Br. J. Clin. Pharmacol..

[65] Nasveld P.E., Edstein M.D., Reid M., Brennan L., Harris I.E., Kitchener S.J., Leggat P.A., Pickford P., Kerr C., Ohrt C. (2010). Randomized, Double-Blind Study of the Safety, Tolerability, and Efficacy of Tafenoquine versus Mefloquine for Malaria Prophylaxis in Nonimmune Subjects. Antimicrob. Agents Chemother..

[66] Price R.N., Simpson J.A., McCarthy J.S. (2017). Halofantrine. Kucers’ the Use of Antibiotics.

[67] Wattanakul T., Ogutu B., Kabanywanyi A.M., Asante K.-P., Oduro A., Adjei A., Sie A., Sevene E., Macete E., Compaore G. (2020). Pooled Multicenter Analysis of Cardiovascular Safety and Population Pharmacokinetic Properties of Piperaquine in African Patients with Uncomplicated Falciparum Malaria. Antimicrob. Agents Chemother..

[68] Dhingra S.K., Redhi D., Combrinck J.M., Yeo T., Okombo J., Henrich P.P., Cowell A.N., Gupta P., Stegman M.L., Hoke J.M. (2017). A Variant PfCRT Isoform Can Contribute to *Plasmodium falciparum* Resistance to the First-Line Partner Drug Piperaquine. mBio.

[69] White N.J., van Vugt M., Ezzet F.D. (1999). Clinical Pharmacokinetics and Pharmacodynamics of Artemether-Lumefantrine. Clin. Pharmacokinet..

[70] Stover K.R., King S.T., Robinson J. (2012). Artemether-Lumefantrine: An Option for Malaria. Ann. Pharmacother..

[71] Ashley E.A., Recht J., White N.J. (2014). Primaquine: The risks and the benefits. Malar. J..

[72] Ganesan S., Chaurasiya N.D., Sahu R., Walker L.A., Tekwani B.L. (2012). Understanding the mechanisms for metabolism-linked hemolytic toxicity of primaquine against glucose 6-phosphate dehydrogenase deficient human erythrocytes: Evaluation of eryptotic pathway. Toxicology.

[73] Frampton J.E. (2018). Tafenoquine: First Global Approval. Drugs.

[74] de Kock M., Tarning J., Workman L., Allen E.N., Tekete M.M., Djimde A.A., Bell D.J., Ward S.A., Barnes K.I., Denti P. (2018). Population Pharmacokinetic Properties of Sulfadoxine and Pyrimethamine: A Pooled Analysis To Inform Optimal Dosing in African Children with Uncomplicated Malaria. Antimicrob. Agents Chemother..

[75] Helsby N., Edwards G., Breckenridge A., Ward S. (1993). The multiple dose pharmacokinetics of proguanil. Br. J. Clin. Pharmacol..

[76] Veenendaal R., Edstein M.D., Rieckmann K.H. (2009). Pharmacokinetics of Chlorproguanil in Man after a Single Oral Dose of Lapudrine^®^. Chemotherapy.

[77] Ashton M., Gordi T., Hai T.N., Van Huong N., Sy N.D., Nieu N.T., Huong D.X., Johansson M., Công L.D. (1998). Artemisinin pharmacokinetics in healthy adults after 250, 500 and 1000 mg single oral doses. Biopharm. Drug Dispos..

[78] Gobbi F., Buonfrate D., Menegon M., Lunardi G., Angheben A., Severini C., Gori S., Bisoffi Z. (2016). Failure of dihydroartemisinin-piperaquine treatment of uncomplicated *Plasmodium falciparum* malaria in a traveller coming from Ethiopia. Malar. J..

[79] Naing C., Racloz V., Whittaker M.A., Aung K., Reid S.A., Mak J.W., Tanner M. (2013). Efficacy and Safety of Dihydroartemisinin-Piperaquine for Treatment of Plasmodium vivax Malaria in Endemic Countries: Meta-Analysis of Randomized Controlled Studies. PLoS ONE.

[80] Li J., Zhang J., Li Q., Hu Y., Ruan Y., Tao Z., Xia H., Qiao J., Meng L., Zeng W. (2020). Ex vivo susceptibilities of Plasmodium vivax isolates from the China-Myanmar border to antimalarial drugs and association with polymorphisms in Pvmdr1 and Pvcrt-o genes. PLoS Negl. Trop. Dis..

[81] Sridapan T., Rattanakoch P., Kijprasong K., Srisutham S. (2024). Drug resistance markers in Plasmodium vivax isolates from a Kanchanaburi province, Thailand between January to May 2023. PLoS ONE.

[82] Shekalaghe S.A., ter Braak R., Daou M., Kavishe R., van den Bijllaardt W., van den Bosch S., Koenderink J.B., Luty A.J.F., Whitty C.J.M., Drakeley C. (2010). In Tanzania, Hemolysis after a Single Dose of Primaquine Coadministered with an Artemisinin Is Not Restricted to Glucose-6-Phosphate Dehydrogenase-Deficient (G6PD A−) Individuals. Antimicrob. Agents Chemother..

[83] Plowe C.V., Kublin J.G., Doumbo O.K. (1998). *P. falciparum* dihydrofolate reductase and dihydropteroate synthase mutations: Epidemiology and role in clinical resistance to antifolates. Drug Resist. Updat..

[84] Nair S., Miller B., Barends M., Jaidee A., Patel J., Mayxay M., Newton P., Nosten F., Ferdig M.T., Anderson T.J.C. (2008). Adaptive Copy Number Evolution in Malaria Parasites. PLOS Genet..

[85] Kidgell C., Volkman S.K., Daily J., Borevitz J.O., Plouffe D., Zhou Y., Johnson J.R., Roch K.G.L., Sarr O., Ndir O. (2006). A Systematic Map of Genetic Variation in *Plasmodium falciparum*. PLoS Pathog..

[86] Cowell A.N., Winzeler E.A. (2019). The genomic architecture of antimalarial drug resistance. Brief. Funct. Genom..

[87] Ashley E.A., Dhorda M., Fairhurst R.M., Amaratunga C., Lim P., Suon S., Sreng S., Anderson J.M., Mao S., Sam B. (2014). Spread of Artemisinin Resistance in *Plasmodium falciparum* Malaria. N. Engl. J. Med..

[88] Rosenthal M.R., Ng C.L. (2020). *Plasmodium falciparum* Artemisinin Resistance: The Effect of Heme, Protein Damage, and Parasite Cell Stress Response. ACS Infect. Dis..

[89] Rahmasari F.V., Asih P.B.S., Dewayanti F.K., Rotejanaprasert C., Charunwatthana P., Imwong M., Syafruddin D. (2022). Drug resistance of *Plasmodium falciparum* and Plasmodium vivax isolates in Indonesia. Malar. J..

[90] Djimde A.A., Makanga M., Kuhen K., Hamed K. (2015). The emerging threat of artemisinin resistance in malaria: Focus on artemether-lumefantrine. Expert Rev. Anti-Infect. Ther..

[91] Windle S.T., Lane K.D., Gadalla N.B., Liu A., Mu J., Caleon R.L., Rahman R.S., Sá J.M., Wellems T.E. (2020). Evidence for linkage of *pfmdr1*, *pfcrt*, and *pfk13* polymorphisms to lumefantrine and mefloquine susceptibilities in a *Plasmodium falciparum* cross. Int. J. Parasitol. Drugs Drug Resist..

[92] Deng C., Huang B., Wang Q., Wu W., Zheng S., Zhang H., Li D., Feng D., Li G., Xue L. (2018). Large-scale Artemisinin–Piperaquine Mass Drug Administration With or Without Primaquine Dramatically Reduces Malaria in a Highly Endemic Region of Africa. Clin. Infect. Dis..

[93] van der Pluijm R.W., Tripura R., Hoglund R.M., Phyo A.P., Lek D., ul Islam A., Anvikar A.R., Satpathi P., Satpathi S., Behera P.K. (2020). Triple artemisinin-based combination therapies versus artemisinin-based combination therapies for uncomplicated *Plasmodium falciparum* malaria: A multicentre, open-label, randomised clinical trial. Lancet.

[94] Ross L.S., Dhingra S.K., Mok S., Yeo T., Wicht K.J., Kümpornsin K., Takala-Harrison S., Witkowski B., Fairhurst R.M., Ariey F. (2018). Emerging Southeast Asian PfCRT mutations confer *Plasmodium falciparum* resistance to the first-line antimalarial piperaquine. Nat. Commun..

[95] Duru V., Witkowski B., Ménard D. (2016). *Plasmodium falciparum* Resistance to Artemisinin Derivatives and Piperaquine: A Major Challenge for Malaria Elimination in Cambodia. Am. J. Trop. Med. Hyg..

[96] Rasmussen C., Alonso P., Ringwald P. (2022). Current and emerging strategies to combat antimalarial resistance. Expert Rev. Anti-Infect. Ther..

[97] Oladipo H.J., Tajudeen Y.A., Oladunjoye I.O., Yusuff S.I., Yusuf R.O., Oluwaseyi E.M., AbdulBasit M.O., Adebisi Y.A., El-Sherbini M.S. (2022). Increasing challenges of malaria control in sub-Saharan Africa: Priorities for public health research and policymakers. Ann. Med. Surg..

[98] Rajneesh, Tiwari R., Singh V.K., Kumar A., Gupta R.P., Singh A.K., Gautam V., Kumar R. (2023). Advancements and Challenges in Developing Malaria Vaccines: Targeting Multiple Stages of the Parasite Life Cycle. ACS Infect. Dis..

[99] Halbroth B.R., Draper S.J., Rollinson D., Stothard J.R. (2015). Chapter One—Recent Developments in Malaria Vaccinology. Advances in Parasitology.

[100] Aly A.S.I., Vaughan A.M., Kappe S.H.I. (2009). Malaria Parasite Development in the Mosquito and Infection of the Mammalian Host. Annu. Rev. Microbiol..

[101] Nunes-Cabaço H., Moita D., Prudêncio M. (2022). Five decades of clinical assessment of whole-sporozoite malaria vaccines. Front. Immunol..

[102] Seder R.A., Chang L.-J., Enama M.E., Zephir K.L., Sarwar U.N., Gordon I.J., Holman L.A., James E.R., Billingsley P.F., Gunasekera A. (2013). Protection against malaria by intravenous immunization with a nonreplicating sporozoite vaccine. Science.

[103] Birkett A.J., Moorthy V.S., Loucq C., Chitnis C.E., Kaslow D.C. (2013). Malaria vaccine R&D in the Decade of Vaccines: Breakthroughs, challenges and opportunities. Vaccine.

[104] Epstein J.E., Tewari K., Lyke K.E., Sim B.K.L., Billingsley P.F., Laurens M.B., Gunasekera A., Chakravarty S., James E.R., Sedegah M. (2011). Live attenuated malaria vaccine designed to protect through hepatic CD8^+^ T cell immunity. Science.

[105] Hoffman S.L., Goh L.M.L., Luke T.C., Schneider I., Le T.P., Doolan D.L., Sacci J., de la Vega P., Dowler M., Paul C. (2002). Protection of humans against malaria by immunization with radiation-attenuated *Plasmodium falciparum* sporozoites. J. Infect. Dis..

[106] Roestenberg M., McCall M., Hopman J., Wiersma J., Luty A.J.F., van Gemert G.J., van de Vegte-Bolmer M., van Schaijk B., Teelen K., Arens T. (2009). Protection against a malaria challenge by sporozoite inoculation. N. Engl. J. Med..

[107] Kublin J.G., Mikolajczak S.A., Sack B.K., Fishbaugher M.E., Seilie A., Shelton L., VonGoedert T., Firat M., Magee S., Fritzen E. (2017). Complete attenuation of genetically engineered *Plasmodium falciparum* sporozoites in human subjects. Sci. Transl. Med..

[108] Vaughan A.M., Wang R., Kappe S.H.I. (2010). Genetically engineered, attenuated whole-cell vaccine approaches for malaria. Hum. Vaccin..

[109] Butler N.S., Vaughan A.M., Harty J.T., Kappe S.H.I. (2012). Whole parasite vaccination approaches for prevention of malaria infection. Trends Immunol..

[110] Stanisic D.I., Good M.F. (2023). Malaria Vaccines: Progress to Date. Biodrugs.

[111] Syed Y.Y. (2022). RTS,S/AS01 malaria vaccine (Mosquirix^®^): A profile of its use. Drugs Ther. Perspect..

[112] Laurens M.B. (2020). RTS,S/AS01 vaccine (Mosquirix™): An overview. Hum. Vaccines Immunother..

[113] de Almeida M.E.M., de Vasconcelos M.G.S., Tarragô A.M., Mariúba L.A.M. (2021). Circumsporozoite Surface Protein-based malaria vaccines: A review. Rev. Inst. Med. Trop. São Paulo.

[114] Stertman L., Palm A.-K.E., Zarnegar B., Carow B., Lunderius Andersson C., Magnusson S.E., Carnrot C., Shinde V., Smith G., Glenn G. (2023). The Matrix-M™ adjuvant: A critical component of vaccines for the 21st century. Hum. Vaccines Immunother..

[115] Datoo M.S., Dicko A., Tinto H., Ouédraogo J.-B., Hamaluba M., Olotu A., Beaumont E., Ramos Lopez F., Natama H.M., Weston S. (2024). Safety and efficacy of malaria vaccine candidate R21/Matrix-M in African children: A multicentre, double-blind, randomised, phase 3 trial. Lancet Lond. Engl..

[116] Sang S., Datoo M.S., Otieno E., Muiruri C., Bellamy D., Gathuri E., Ngoto O., Musembi J., Provstgaard-Morys S., Stockdale L. (2023). Safety and immunogenicity of varied doses of R21/Matrix-M™ vaccine at three years follow-up: A phase 1b age de-escalation, dose-escalation trial in adults, children, and infants in Kilifi-Kenya. Wellcome Open Res..

[117] Neafsey D.E., Juraska M., Bedford T., Benkeser D., Valim C., Griggs A., Lievens M., Abdulla S., Adjei S., Agbenyega T. (2015). Genetic Diversity and Protective Efficacy of the RTS,S/AS01 Malaria Vaccine. N. Engl. J. Med..

[118] Su X., Zhang C., Joy D.A. (2020). Host-Malaria Parasite Interactions and Impacts on Mutual Evolution. Front. Cell. Infect. Microbiol..

[119] Tajudeen Y.A., Oladipo H.J., Yusuff S.I., Abimbola S.O., Abdulkadir M., Oladunjoye I.O., Omotosho A.O., Egbewande O.M., Shittu H.D., Yusuf R.O. (2024). A landscape review of malaria vaccine candidates in the pipeline. Trop. Dis. Travel Med. Vaccines.

[120] Duffy P.E. (2022). Current approaches to malaria vaccines. Curr. Opin. Microbiol..

[121] Goodier M.R., Wolf A.-S., Riley E.M. (2020). Differentiation and adaptation of natural killer cells for anti-malarial immunity. Immunol. Rev..

[122] Patarroyo M.A., Molina-Franky J., Gómez M., Arévalo-Pinzón G., Patarroyo M.E. (2020). Hotspots in Plasmodium and RBC Receptor-Ligand Interactions: Key Pieces for Inhibiting Malarial Parasite Invasion. Int. J. Mol. Sci..

[123] Chitnis C.E., Sharma A. (2008). Targeting the Plasmodium vivax Duffy-binding protein. Trends Parasitol..

[124] Weiss G.E., Traore B., Kayentao K., Ongoiba A., Doumbo S., Doumtabe D., Kone Y., Dia S., Guindo A., Traore A. (2010). The *Plasmodium falciparum*-Specific Human Memory B Cell Compartment Expands Gradually with Repeated Malaria Infections. PLOS Pathog..

[125] White N.J., Pukrittayakamee S., Hien T.T., Faiz M.A., Mokuolu O.A., Dondorp A.M. (2014). Malaria. Lancet Lond. Engl..

[126] Murray C.J.L., Rosenfeld L.C., Lim S.S., Andrews K.G., Foreman K.J., Haring D., Fullman N., Naghavi M., Lozano R., Lopez A.D. (2012). Global malaria mortality between 1980 and 2010: A systematic analysis. Lancet Lond. Engl..

[127] Acharya P., Garg M., Kumar P., Munjal A., Raja K.D. (2017). Host–Parasite Interactions in Human Malaria: Clinical Implications of Basic Research. Front. Microbiol..

[128] Frimpong A., Kusi K.A., Ofori M.F., Ndifon W. (2018). Novel Strategies for Malaria Vaccine Design. Front. Immunol..

[129] Kogan F., Kogan F. (2020). Malaria Burden. Remote Sensing for Malaria: Monitoring and Predicting Malaria from Operational Satellites.

[130] Roberts T., Cohn J., Bonner K., Hargreaves S. (2016). Scale-up of Routine Viral Load Testing in Resource-Poor Settings: Current and Future Implementation Challenges. Clin. Infect. Dis..

[131] Ruckstuhl L., Lengeler C., Moyen J.M., Garro H., Allan R. (2017). Malaria case management by community health workers in the Central African Republic from 2009–2014: Overcoming challenges of access and instability due to conflict. Malar. J..

[132] Ishtiaq F. (2021). Ecology and Evolution of Avian Malaria: Implications of Land Use Changes and Climate Change on Disease Dynamics. J. Indian Inst. Sci..

[133] Cowell A.N., Winzeler E.A. (2019). Advances in omics-based methods to identify novel targets for malaria and other parasitic protozoan infections. Genome Med..

[134] Carolino K., Winzeler E.A. (2020). The antimalarial resistome—Finding new drug targets and their modes of action. Curr. Opin. Microbiol..

[135] Abuga K.M., Jones-Warner W., Hafalla J.C.R. (2021). Immune responses to malaria pre-erythrocytic stages: Implications for vaccine development. Parasite Immunol..

[136] Stutzer C., Richards S.A., Ferreira M., Baron S., Maritz-Olivier C. (2018). Metazoan Parasite Vaccines: Present Status and Future Prospects. Front. Cell. Infect. Microbiol..

[137] Franke-Fayard B., Marin-Mogollon C., Geurten F.J.A., Chevalley-Maurel S., Ramesar J., Kroeze H., Baalbergen E., Wessels E., Baron L., Soulard V. (2022). Creation and preclinical evaluation of genetically attenuated malaria parasites arresting growth late in the liver. Npj Vaccines.

[138] Burt A., Coulibaly M., Crisanti A., Diabate A., Kayondo J.K. (2018). Gene drive to reduce malaria transmission in sub-Saharan Africa. J. Responsible Innov..

